# Case report: Cement entrapped in the inferior vena cava filter after pedicle screw augmentation

**DOI:** 10.3389/fcvm.2022.892025

**Published:** 2022-09-29

**Authors:** Xinqiang Han, Yongzhen Zhang, Zhu Wang, Mengpeng Zhao

**Affiliations:** Department of Interventional Medicine and Vascular, Binzhou Medical University Hospital, Binzhou, China

**Keywords:** vertebroplasty, bone cement, inferior vena cava, inferior vena cava filter, complication

## Abstract

**Background:**

Cement leakage into the inferior vena cava (IVC) is one of the most common complications associated with cement vertebroplasty, and can lead to potentially life-threatening complications such as pulmonary cement embolism (PCE). Implantation of an IVC filter is effective in the prevention of fatal pulmonary embolism. Here, we present an extremely rarely case of cement entrapped in an IVC filter after pedicle screw augmentation, and discuss all similar cases reported in the literature.

**Case presentation:**

A 70-year-old female presented with significant back and lower extremities pain and was unable to walk. MRI of the lumbar spine revealed an osteoporotic compression fracture of the L1–L3. She underwent cement-augmented pedicle screws implanted at the L1 and L3 vertebral bodies. A retrievable IVC filter was implanted due to the presence of calf vein thrombosis before cement vertebroplasty. Cement leaked into the IVC and was trapped by the filter, rendering the filter unretrievable using a conventional method. The asymptomatic patient received rivaroxaban 20 mg daily for anticoagulant postoperatively and lifelong anticoagulation was administered to prevent secondary IVC and cemented filter thrombosis.

**Methods:**

A literature search was conducted utilizing the PUBMED/MEDLINE using the following terms: “vertebroplasty,” “complication,” “bone cement,” and “inferior vena cava (IVC),” or “inferior vena cava (IVC) filter.” All relevant articles published in English or in other languages with English abstracts since 1962 were included.

**Results:**

A total of 36 articles were retrieved according to the search strategy. Only 6 out of these 36 studies contained information regarding the inferior vena cava filter and cement. Of the patients, 85.7% (36/42) reported in the literature whose gender was known were female and 14.3% were male. 28.5% (45/158) patients with pulmonary arterial and cardiovascular complications.

**Conclusion:**

Cement embolization occurring in the IVC filter is rare. Accurate knowledge about the lumbar vertebral venous anatomy and skillful operation during vertebral cementoplasty should be required in clinical practice.

## Introduction

Bone cement has been widely injected into diseased or fractured vertebral bodies to provide an expected increase in the stabilization and strength of the vertebral and pedicle screws, and is especially suitable for the management of osteoporotic compression fractures (1). However, spillage of cement frequently occurs in the process of vertebroplasty and may result in serious sequelae. Cement leakage into the venous plexus is the most frequent complication, and can lead to severe and potentially life-threatening complications such as pulmonary cement embolism (PCE) or myocardial perforation (2). Here, we present a case that underwent cement augmentation for improving fixation of pedicle screws, in which the cement leaked into the peripheral venous system causing large masses extending to the IVC that were eventually captured in the previously implanted IVC filter. This case study is significant because it illustrates the rare occurrence of cemented filter, making clinicians aware of the potential possibility of cement leakage into the IVC in patients with cement-based augmentation of vertebroplasty. We discuss this case and all similar cases reported in the literature.

## Case presentation

There was a 70-year-old female patient who had significant back and lower extremities pain and was unable to walk. An outpatient MRI of the lumbar spine showed an osteoporotic compression fracture of the L1–L3. She had cement- (polymethylmethacrylate) augmented pedicle screws implanted at the L1 and L3 vertebral bodies after hospital admission. A total of 5.8 ml of “tooth-paste-like” bone cement was injected to provide stabilization and pain relief within 5 min after mixing, and the procedure was performed under the guidance of C-arm X-ray fluoroscopy. Before cement vertebroplasty, a retrievable IVC filter (Aegisy, LifeTech, Shenzhen, China) was implanted into the IVC to prevent pulmonary embolism (PE) since the routine preoperative ultrasound revealed an isolated distal deep vein thrombosis in the vein of her left calf. The patient received rivaroxaban 20 mg daily for anticoagulant postoperatively.

**FIGURE 1:**
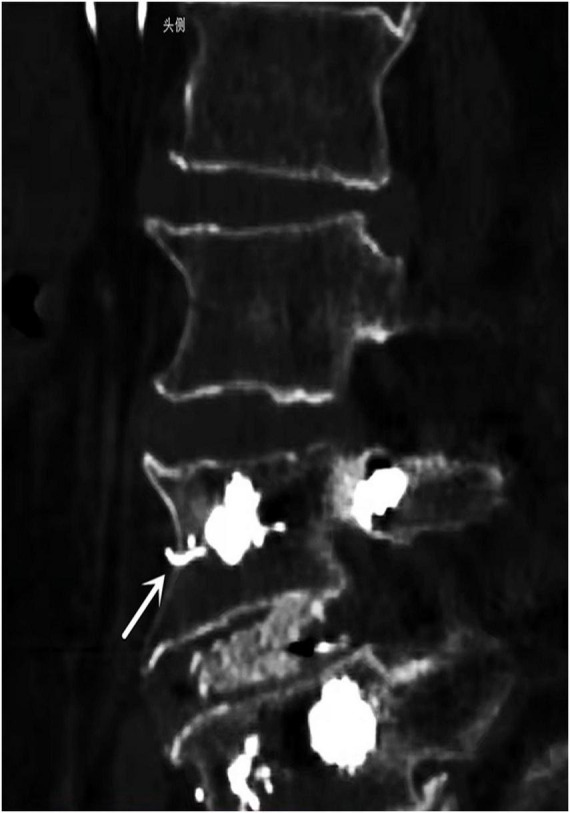
CT scans revealed strip-like high density cement extravasation (arrows) in vertebra bone.

**FIGURE 2:**
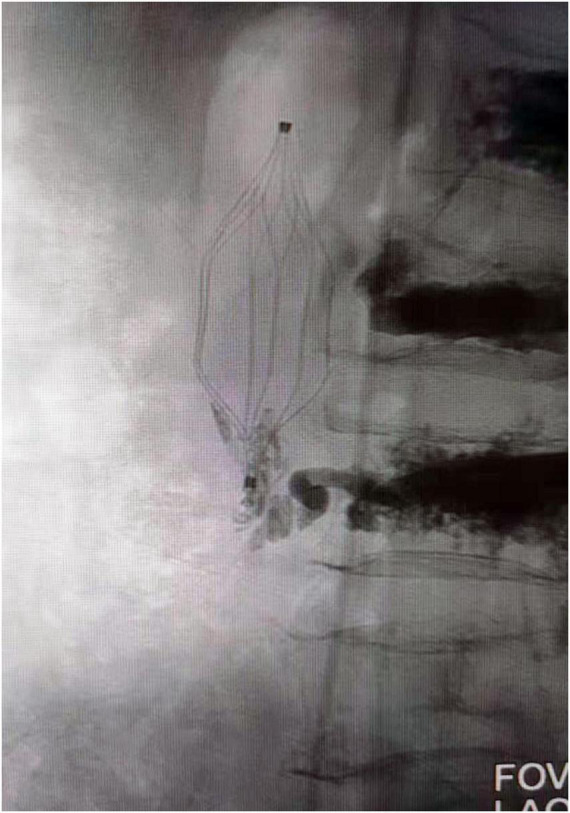
Venography showed cement attached to the IVC filter hook and trapped in the filter. IVC, inferior vena cava.

Thereafter, X-ray examination showed paravertebral venous cement leak and cement entry into the IVC, resulting in the deposition of a cement cast in the IVC filter, and CT images demonstrating cement leaking out of the vertebral body (Figure 1). An inferior vena cavography prior to retrieval of the filter showed that cement had migrated into the IVC and attached to the caval wall at the level of the IVC filter tip and was trapped within the filter (Figure 2), resulting in the IVC filter failing to be retrieved in the conventional way.

Bone cement injection was performed by spinal surgeons with more than 10 years of clinical experience, and cavography and DSA image judgment were performed by interventional radiologists with more than 10 years of clinical experience. The asymptomatic patient continued with rivaroxaban 20 mg daily and was discharged from the hospital, and close follow-up and lifelong anticoagulation was administered to prevent secondary IVC and cemented filter thrombosis.

## Methods

A literature search was conducted utilizing the PUBMED/MEDLINE using the following terms: “vertebroplasty,” “complication,” “bone cement,” and “inferior vena cava (IVC),” or “inferior vena cava (IVC) filter.” All relevant articles published in English or in other languages with English abstracts since 1962 were included.

## Results

The literature search yielded 36 articles whose clinical reports contained information regarding the coexistence of cement and inferior vena cava or inferior vena cava filter. Of these patients, 85.7% (36/42) of individuals whose gender was known were female (*n* = 36) and 14.3% (6/42) were male (*n* = 6). Cement leakage into the IVC that primarily resulted in pulmonary arterial and cardiovascular complications despite the majority of the patients being asymptomatic accounted for 45 of 158 cases. Only 6 out of these 36 studies contained information regarding the inferior vena cava filter and cement.

Our personal experience includes one unpublished female patient seen and followed-up at our institutions. In addition, another earlier and similar case to this has been accepted for publication in a future issue of CardioVascular and Interventional Radiology, but has not been fully edited. The clinical features of those 36 previously reported are presented in Tables 1, 2.

**TABLE 1:** Cases of cement leakage extended into the inferior vena cava reported in literature.

Authors	Year	Numbers	Gender	Outcome
Prater et al. (15)	2021	1	F	IVC filter trapped
Hu et al. (1)	2021	1	F	PCE and iliac vein thrombus
David et al. (28)	2021	1	F	PCE
Kong et al. (9)	2019	12	11F/1M	1 cardiac embolus and 11 PCE
Izumi et al. (29)	2019	1	F	PCE and shock
Ishak et al. (24)	2019	14	N/A	PCE (4)
Frenk et al. (30)	2019	1	M	Stent placement and anchor to IVC
Yuan et al. (10)	2018	1	F	PCE
Saranteas et al. (7)	2018	2	1F/1M	1 died/1 cardiac embolus
Park et al. (31)	2018	1	F	Intra-cardiac embolism
Majunke et al. (32)	2018	1	F	PCE and successful retrieval
Isaak et al. (33)	2018	1	N/A	PCE
Elens et al. (34)	2018	1	F	Anchor to IVC
Suwan et al. (35)	2017	1	N/A	Anchor to IVC
Janssen et al. (8)	2017	94	N/A	13 PCE (2 died)
Riesner et al. (2)	2016	2	N/A	No PCE
Shen et al. (36)	2015	1	F	PCE and Cardiac perforation
Edwards et al. (16)	2015	1	F	IVC filter trapped
Schmid et al. (37)	2014	1	M	PCE
Lee et al. (38)	2014	1	F	Cardiac tamponade and died
Vallabhajosyula et al. (39)	2013	1	F	PCE
Li et al. (17)	2013	1	F	IVC filter trapped
Sun et al. (40)	2012	1	F	IVC thrombosis
Schulz et al. (41)	2012	3	N/A	PCE
Kim et al. (42)	2012	1	M	IVC thrombosis and PCE
Czesla et al. (43)	2012	1	F	PCE and Cardiac perforation
Dash et al. (44)	2011	1	F	PCE and Right atrial-IVC thrombus
Agko et al. (14)	2010	1	F	IVC filter trapped
Kim et al. (11)	2009	2	1M	PCE
Athreya et al. (18)	2009	1	F	IVC filter trapped
Kao et al. (27)	2008	1	F	IVC thrombosis and PCE
Lim et al. (45)	2007	1	F	PCE and intraatrial thrombus
Herbstreit et al. (19)	2006	1	F	IVC filter trapped and cavotomy
Baumann et al. (46)	2005	1	F	PCE
Prymka et al. (47)	2003	1	F	N/A
Padovani et al. (48)	1999	1	F	PCE

IVC, inferior vena cava; PCE, pulmonary cement embolism.

## Discussion

Cement augmentation with polymethylmethacrylate is a reliable method for increasing the compressive strength of vertebral bodies, and it is an accepted treatment for osteoporotic compression fracture and reinforcement of vertebral pedicle screws fixation. Perivertebral cement leakage is a frequently reported complication following pedicle screw augmentation and it could potentially be a life-threatening condition; the leaked cement may migrate into IVC and pulmonary arterial circulation with varying incidence rates of 0.9–26% (3–5). However, it is often the case that there were no routine chest CT examinations before or after vertebroplasty. The true incidence of PCE is unknown and may even be underestimated. In addition, the mortality data related to cement leakage was not systematically documented in the literature (6, 7). Although a number of patients have bone cement leakage into the azygos vein or IVC, most cases remain asymptomatic (8). Despite this, some catastrophic outcomes, such as fatal PCE, cardiovascular collapse, and myocardial perforation, have still been documented in the literature (6, 9, 10). Kim found that cement leakage into the IVC showed a statistically significant correlation with PCE (*p* = 0.03) (11).

It is well known that implantation of IVC filter is effective in the prevention or reduction of the risk of fatal PE in patients with high risk of lower extremity deep vein thrombosis (12). In rare cases, some IVC filters have also been used as a prevention strategy against cement emboli in IVC, which may migrate into the pulmonary circulation (13, 14). However, it appears to be quite rare for leaked cement to become entrapped in an IVC filter. So far as we know, only six cases of cemented filters have previously been reported in the literature (Table 2) (14–19). In our case, cement leaked into the IVC and was trapped by the filter, preventing cement cast migration to the pulmonary arterial circulation. However, it is extremely difficult to retrieve the cemented filter into the outer sheath by utilizing conventional endovascular procedures. Management guidelines vary because of the rare nature of this event. In our case, careful observation and long-term anticoagulation were recommended. Nevertheless, with secondary events, IVC foreign bodies serve as a nidus for thromboembolism and should be taken into consideration.

**TABLE 2:** Cases of cement trapped by inferior vena cava filter reported in literature.

Authors	Year	Age (y)	Gender	Filter brand	Outcome
Prater et al. (15)	2021	77	F	N/A	Unremoved
Edwards et al. (16)	2015	45	F	N/A	Removed
Li et al. (17)	2013	58	F	OptEase	Unremoved
Agko et al. (14)	2010	51	F	Greenfield	Removed
Athreya et al. (18)	2009	61	F	Guenther Tulip	Removed
Herbstreit et al. (19)	2006	66	F	N/A	Cavotomy

Therefore, do we need to implant an IVC filter to prevent PCE before vertebral cementoplasty? The only randomized trial regarding pulmonary complications during the cementoplasty procedure is the VERTOS II trial, and 26% (14/54) of the patients with PCE were confirmed by chest CT scans after the vertebral cementoplasty despite none of the patients having related symptoms due to the small size of the cement embolus and its random distribution in the pulmonary vascular endings (20). Among the 158 patients with cement leakage into the IVC after vertebral cementoplasty of all relevant retrieved articles by 2021 in our study, 45 (28.5%) had cardiopulmonary complications despite the majority presenting as mild cases, but severe complications such as cardiac perforation and circulatory failure did occur (Table 1). However, some cardiopulmonary complications were not well documented, and the incidence appeared to be underestimated. Apparently, the risk–benefit ratio for IVC filter implantation is far from certain. Therefore, decisions need to be made on a case-by-case basis.

**FIGURE 3:**
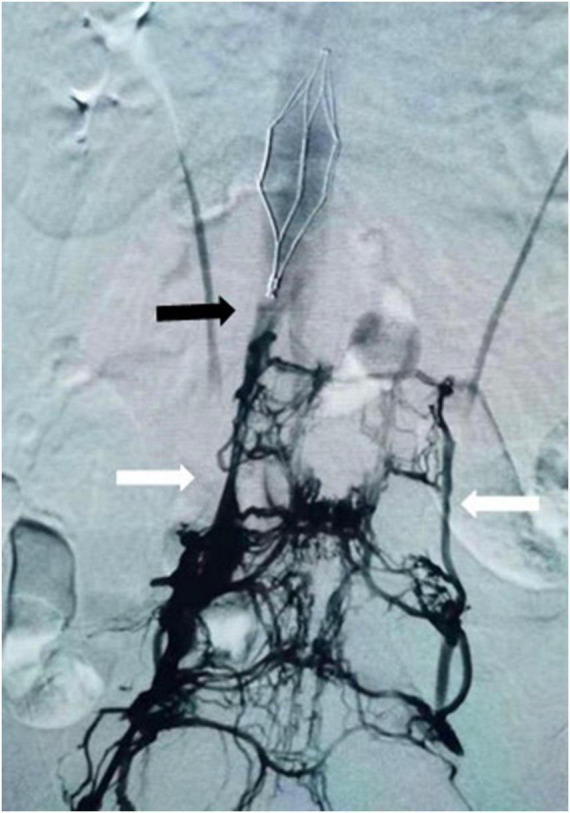
Venography showed collateral drainage veins between inferior vena cava (black arrow) and paravertebral venous (white arrow).

The reasons why cement embolization occurs in the IVC during cementoplasty procedure have been much discussed in the literature. It is known to all that lumbar vertebral veins enter the IVC at L1-L5 vertebral levels, and numerous connections exist with basivertebral vein and segmental vein (Figure 3) (5, 21). Iwanaga found that latex or air can flow into the IVC at the internal/external vertebral plexus through anatomical location (5). This is the anatomic risk factor for the occurrence of cement venous leakage. However, it should be noted that there may be other reasons, such as incompletely polymerizing cement, the proximity of the needle to the vertebral venous plexus, or the higher volume and faster pushing of the cement (22). Additionally, intraoperative X-ray fluoroscopy may also be helpful to reduce cement leakage (23). But Ishak, who found that 55 of 86 patients had venous cement leakage despite 52 of them having no symptoms, suggested that using CT navigation for screw placement did not reduce the cement leakage risk (24). This may be in part related to a non-radiologist operator with C-arm fluoroscopy (11). Phillips found that injection of contrast medium into the vertebral body could also predict and reduce the occurrence of bone cement leakage (25). Post-procedure chest CT scans may be useful in guiding early diagnosis and treatment. In all literature reports, the proportion of females is significantly higher than that of males. It is not clear why cement leakage into the IVC is more likely to occur in females, although Zhan found no significant association between gender and cement leakage after vertebroplasty or kyphoplasty in a recent systematic review (26).

## Conclusion

These interesting cases illustrate that the IVC filter could capture cement that leaks into the IVC and prevent fatal pulmonary arterial and cardiovascular complications. The present study, as with any other (27), contributed to making clinicians aware of the potential occurrence of cement leakage into the IVC during vertebroplasty procedures. In other words, surgeons should be aware of the possibility of cement leakage when patients develop clinical symptoms of PE, such as decreased blood pressure, tachycardia, and dyspnea.

## Data availability statement

The raw data supporting the conclusions of this article will be made available by the authors, without undue reservation.

## Ethics statement

Written informed consent was obtained from the individual(s) for the publication of this case report and the publication of any potentially identifiable images or data included in this article.

## Author contributions

XH, YZ, and MZ: study concept, acquisition of data and figures, and writing of the manuscript. MZ and ZW: critical revision of manuscript for intellectual content. All authors cared for the patient and contributed to writing of the report.
